# Type 2 diabetes and COPD: treatment in the right healthcare setting? An observational study

**DOI:** 10.1186/s12875-021-01424-w

**Published:** 2021-04-20

**Authors:** R. P. Willink, Rimke C. Vos, I. Looijmans-van den Akker, Huberta E. Hart

**Affiliations:** 1 Department of General Practice, Julius Center for Health Sciences and Primary Care, University Medical Center Utrecht, Utrecht University, Utrecht, the Netherlands; 2 Department of Public Health and Primary Care, Leiden University Medical Center, LUMC-Campus, The Hague, the Netherlands; 3 Leidsche Rijn Julius Healthcare Centers, Utrecht, the Netherlands

**Keywords:** Primary health care, Diabetes mellitus, Type 2, Pulmonary disease, Chronic obstructive

## Abstract

**Background:**

Type 2 diabetes (T2DM) and COPD are chronic medical conditions, for which patients need lifelong healthcare. The aim of this study is to examine in which healthcare setting patients with T2DM and COPD receive their care, and if this is the correct healthcare setting according to guidelines.

**Method:**

T2DM and COPD patients from five primary care practices were included. Data concerning healthcare setting and patient- and clinical characteristics were extracted from the electronic medical records. Patient profiles treated in primary care were compared with the profiles of those treated in secondary care. In patients treated in secondary care we evaluated whether treatment allocation was according to the guidelines and if back-referral to primary care should take place.

**Results:**

Of the T2DM and COPD patients 7.6% and 29.6% respectively, were treated in secondary care, and 72.7% respectively 31.4% of these were according to the guideline. T2DM patients treated in primary care were older (63 versus 57 years, *p* < 0.01, had a shorter diabetes duration (8 versus 11 years, *p* < 0.01) and lower HbA1c (53.0 versus 63.5 mmol/l, *p* < 0.01) than those treated in secondary care. Those with COPD treated in primary care used less inhalation medication (75.2 versus 90.1%, *p* < 0.01) and had better spirometry results (67.39 versus 57.53 FEV_1_%pred, *p* < 0.01).

**Conclusion:**

The majority of the patients with T2DM and COPD were correctly treated in primary care and on average patients with a better health condition were treated in primary care.. Also, those who were treated in secondary care were most of the time treated in the correct treatment setting according to the guidelines.

**Supplementary Information:**

The online version contains supplementary material available at 10.1186/s12875-021-01424-w.

## Background

Diabetes Mellitus (DM) and Chronic Obstructive Pulmonary Disease (COPD) are high prevalent chronic conditions; in 2019 over 1.1 million people in the Netherlands were diagnosed with DM [1], and almost 600.000 with COPD [2]. In the Netherlands the majority of patients with type 2 Diabetes Mellitus (T2DM) and with COPD are, according to the guidelines, treated in primary care [3, 4]. In T2DM this care is important for the prevention of microvascular (retinopathy, nephropathy, and neuropathy) and macrovascular complications (among others stroke and myocardial infarction) [5, 6]. In COPD care focusses on smoking cessation, reduction of exacerbations, decreasing dyspnoea, slowing disease progression, improving health status and reducing mortality [7, 8]. Both chronic conditions represent an enormous global health burden. For DM, the estimated direct global health expenditure in 2019 was USD 670 billion and is expected to grow [9]. The direct cost of COPD is 6% of total healthcare spending (€38.6 billion annually) in the European Union and accounts for 56% of the total cost of treating respiratory diseases [10]. In the Netherlands only a small percentage of the total budget for DM care is spent in the primary care setting. From the total COPD budget 6% is allocated to primary care and 9.1% to secondary care [11]. The rest of the budgets are, amongst other, spent on eHealth devices and homecare. While from a cost-effectiveness and a patients perspective it is important to provide healthcare in primary care as much as possible.

Currently, in The Netherlands the government supports a project to provide healthcare close to patients’ homes and only when necessary in secondary care, ‘The right care in the right place’ [12]. This national project is set up in order to keep healthcare accessible for all patients, preventing it to get too expensive and to blend care whenever possible with E-health. In the current study we assessed the care setting of patients with T2DM and COPD from five primary care practices in the Netherlands, if they were allocated to the right setting according to the Regional Transmural Agreements (RTA), and also if there was room for improvement. In addition, we explored if the patient profiles from primary care differ from those in secondary care.

## Method

### Study design and setting

Data for this observational study were obtained from electronic medical records from five primary care practices in the center of the Netherlands. Considering this is an observational study using pseudonymized routine care data and that the patients had consented before on using their routine care data for research purposes, approval from the Medical Ethical Committee was not obligated.

### Data collection

All patients within the five primary care practices with an International Classification of Primary Care (ICPC) code for T2DM (T90.02) and/or COPD (R95) were included. In august 2019 data were extracted from the electronic medical records (over the period January 1^st^,2017 until July 26^th^, 2019).

For patients with T2DM and COPD the treatment setting was determined: treatment in primary care, treatment in secondary care or no care at all. For T2DM and COPD patients who were treated in secondary care the reason for referral was extracted and compared with the RTA for T2DM and COPD (see Additional file 1). The RTA for T2DM provides clear criteria when to refer a patient. For COPD patients the treatment setting was determined differently. When COPD patients treated in secondary care did not meet the criteria for treatment in primary care, they were classified as correctly treated in secondary care. For T2DM and COPD patients treated in secondary care it was assessed if a specialist report was received during the study period. If there was no specialist letter, routine care data from the electronic medical records were used.

#### Referral and follow up of healthcare in secondary care

In the Netherlands most patients with T2DM and COPD are treated in primary care [4]. National and regional agreements have been developed to define the indications for referral of patients from primary to secondary care. When referring a patient to the hospital the GP sends all relevant patient- and disease information to the specialist in secondary care. This referral is accompanied by a detailed clinical question and a suggestion for the follow-up care. During the treatment in secondary care the treating specialist yearly sends a report to the GP to inform the GP about the treatment provided in secondary care.

### Patient profiles

#### Type 2 diabetes specific characteristics

Data were extracted from the electronic medical records on patient characteristics (age and sex), disease duration, systolic blood pressure, glycosylated hemoglobin (HbA1c), LDL-cholesterol, estimated glomerular filtration rate, albumin/creatinine ratio in urine, body mass index, diabetic complications, glucose lowering medication, statin use and lifestyle advise.

Disease duration was defined in years and calculated as the duration until 2018. Data on the microvascular complications retinopathy, nephropathy and neuropathy were collected. Retinopathy was defined as retinopathy seen with fundoscopy in one or two eyes. Nephropathy was defined as an eGFR lower than 60 and/or an albumin/creatinine ratio in urine from 3.0 or higher. For the estimated glomerular filtration rate the Chronic Kidney Disease Epidemiology Collaboration formula was used. Neuropathy was defined as a decreased sensation of the monofilament on one or both feet and/or a score from two or higher on the modified Sims classification. Data on the macrovascular complications angina pectoris (ICPC K74), myocardial infarction (ICPC K75), ischemic heart disease without angina (ICPC K76), transient cerebral incident (ICPC K89), cerebral infarction (ICPC K90.03) and intermittent claudication (ICPC K92.1) were extracted from the electronic medical records. Both micro- and macrovascular complications were defined as the presence of one or more complications per category.

#### COPD specific characteristics

Patient characteristics (age and sex), systolic blood pressure, use of inhalation medication, spirometry results, smoking status, body mass index, Clinical COPD Questionnaire (CCQ) and Medical Research Council (MRC) dyspnea scale scores were extracted from the electronic medical records.. The CCQ is a questionnaire used to establish the health status of COPD patients. It consists of three domains: symptom status (4 items), functional status (4 items) and mental status (2 items). The outcome is a sum of all domains ranging from zero to six, with a higher outcome indicating a lower health status [13]. The MRC dyspnea scale is a scale to establish how much dyspnea patients experience, it ranges from one to five, with a higher score indicating more dyspnea [14]. Spirometry results are represented by the percentage of expected on the forced expiratory volume in one second (FEV1%pred).

#### Chronic disease related characteristics

Polypharmacy was defined as the prescription of five or more chronic medications per patient [15]. Frailty of patients was determined according to the Frailty Index [16]. Patients were considered frail when the Frailty Index was higher than 0.2. Data on polypharmacy and frailty were only available for patients 60 years and older. Multimorbidity was defined as the presence of two or more chronic conditions selected from a list from the Netherlands institute for health services research (NIVEL) [17].

### Statistical analysis

Descriptive statistics were performed using IBM SPSS Statistics (version 25.0, IBM Corporation, Armonk, New York, USA). If more than one measure of the same determinant was present in the study period, the average of the measures was calculated and used for further analysis. Difference in continues characteristics between patients treated in primary care and those treated in secondary care were determined by using the Independent T-test or Mann–Whitney U test when appropriate. Differences in categorical variables were determined by using the χ^2^-test or Fisher exact test when appropriate. A p-value of less than 0.05 was considered to be statistically significant.

## Results

### Study population

In 2018 a total of 43,488 patients was enlisted in the five primary care practices, among which 1,439 T2DM patients (prevalence 3.3%) and 409 COPD patients (prevalence 0.9%). Of the T2DM patients 1,329 (92.4%) were treated in primary care and 110 patients (7.6%) in secondary care. Of the COPD patients 270 (66.0%) were treated in primary care and 121 patients (29.6%) in secondary care. There were 18 patients (4.4%) who did not receive any care at all for COPD, these were excluded from further analysis.

### Patient profile

#### Type 2 diabetes

T2DM patients treated in primary care were compared with those treated in secondary care. Primary care patients were older (63 versus 57 years, *p* < 0.01), had a shorter diabetes duration (8 (IQR 9) versus 11 (IQR 12) years, *p* < 0.01), and a lower body mass index (28.73 versus 32.00, *p* = 0.016).

Their HbA1c was lower (53.00 versus 63.50 mmol/l, *p* < 0.01), as was their median albumin/creatinine ratio (0.70 versus 2.60 mg/mmol, *p* < 0.01). The estimated glomerular filtration rate was higher in primary care patients (83.63 versus 79.31 ml/min/1,73m^2^, *p* = 0.05) (Table 1). Overall, primary care patients had less macrovascular complications (19.9 versus 28.2%, *p* = 0.040) and microvascular complications (31.9 versus 48.2%, *p* < 0.001) than patients in secondary care. Primary care patients were more often treated with lifestyle advice only (28.0 versus 7.3%, *p* < 0.001), used less insulin (14.5 versus 72.7%, *p* < 0.001), less glucagon-like peptide-1 agonists (0.9 versus 5.5%, *p* = 0.002) and less sodium-glucose transport protein 2 inhibitors (0.8 versus 4.5%, *p* = 0.004) than those treated in secondary care. So patients with better disease controle were treated in the primary care.

**Table 1:** Profile of the T2DM patients in primary and secondary care

**Diabetes Mellitus type 2**			
	**Primary care (*n* = 1329)**	**Secondary care (*n* = 110)**	***p*-value**
**Sex**			
*Male, n (%)*	741 (55.8)	63 (57.3)	0.76
**Age (mean, range) in years**	63 (20–95)	57 (22–83)	0.001
**Diabetes duration in years (median, IQR)**	8.00 (9.00)	11.00 (12.00)	< 0.001
*Missing, n*	18	19	
**Systolic blood pressure (mmHg, mean ± SD)**	134 ± 15.81	136 ± 17.40	0.36
*Missing, n*	87	13	
**HbA1c (mmol/mol, median, IQR)**	53.00 (14.00)	63.50 (22.50)	< 0.001
*Missing, n*	99	19	
**LDL-cholesterol (mmol/l, mean ± SD)**	2.33 ± 0.84	2.40 ± 0.88	0.51
*Missing, n*	162	47	
**eGFR (ml/min/1,73m**^**2**^** mean ± SD)**	83.63 ± 19.82	79.31 ± 21.90	0.046
*Missing, n*	133	18	
**ACR (mg/mmol, median, IQR)**	0.70 (1.60)	2.60 (4.65)	0.002
*Missing, n*	205	57	
**BMI (kg/m**^**2**^**, median, IQR)**	28.73 (6.16)	32.00 (7.68)	0.016
*Missing, n*	111	65	
**Frailty**			0.11
60 years or older (n)	775	51	
*Yes, n (%)*	489 (63.1)	38 (74.5)	
*No, n (%)*	138 (17.8)	5 (9.8)	
*Missing, n*	148 (19.1)	8 (15.7)	
**Polypharmacy**			
60 years or older (n)	775	51	0.069
*Yes, n (%)*	480 (61.9)	38 (74.5)	
*No, n (%)*	149 (19.2)	5 (9.8)	
*Missing, n(%)*	146 (18.8)	8 (15.7)	
**Medication**			
**No T2DM medication, only lifestyle advise**			
*Yes, n (%)*	372 (28.0)	8 (7.3)	< 0.001
***Statin***			
*Yes, n (%)*	888 (66.8)	64 (58.2)	0.075
***Metformin***			
*Yes, n (%)*	959 (72.2)	72 (65.5)	0.15
***Insulin***			
*Yes, n (%)*	193 (14.5)	80 (72.7)	< 0.001
***GLP1 receptor agonist***			
*Yes, n (%)*	12 (0.9)	6 (5.5)	0.002
***SU deritative***			
*Yes, n (%)*	466 (35.1)	32 (29.1)	0.21
***Oral combination preparation***			
*Yes, n (%)*	3 (0.2)	0 (0.0)	1.00
***Pioglitazone***			
*Yes, n (%)*	2 (0.2)	0 (0.0)	1.00
***Repaglinide***			
*Yes, n (%)*	1 (0.1)	1 (0.9)	0.15
***SGLT2 inhibitor***			
*Yes, n (%)*	10 (0.8)	5 (4.5)	0.004
***DPP4 inhibitor***			
*Yes, n (%)*	49 (3.7)	5 (4.5)	0.60
**Complications**			
**Macrovascular**			
*Yes, n (%)*	265 (19.9)	31 (28.2)	0.040
***Angina pectoris***			
*Yes, n (%)*	80 (6.0)	11 (10.0)	0.099
***Myocardial infarction***			
*Yes, n (%)*	100 (7.5)	14 (12.7)	0.052
***Ischemic heart disease without angina***			
*Yes, n (%)*	33 (2.5)	1 (0.9)	0.51
***Transient cerebral incident***			
*Yes, n (%)*	35 (2.6)	5 (4.5)	0.23
***Cerebral infarction***			
*Yes, n (%)*	9 (0.7)	5 (4.5)	0.003
***Intermittent claudication***			
*Yes, n (%)*	40 (3.0)	5 (4.5)	0.39
**Microvascular**			
*Yes, n (%)*	424 (31.9)	53 (48.2)	< 0.001
*No, n (%)*	867 (65.2)	41 (37.3)	
*Missing, n (%)*	38 (2.9)	16 (14.5)	
***Diabetic retinopathy***			
*Yes, n (%)*	82 (6.2)	28 (25.5)	< 0.001
*No, n (%)*	1140 (85.8)	82 (74.5)	
*Missing, n(%)*	107 (8.1)	0 (0.0)	
***Neuropathy***			
*Yes, n (%)*	114 (8.6)	11 (10.0)	< 0.001
*No, n (%)*	951 (71.6)	99 (90.0)	
*Unclear, n (%)*	19 (1.4)	0 (0.0)	
*Missing, n(%)*	245 (18.4)	0 (0.0)	
***Nephropathy***			
*Yes, n (%)*	275 (20.7)	36 (32.7)	0.001
*No, n (%)*	939 (70.7)	58 (52.7)	
*Missing, n(%)*	115 (8.7)	16 (14.5)	
**Multimorbidity**			
*Yes, n (%)*	890 (67.0)	75 (68.2)	0.82
*No, n (%)*	350 (26.3)	27 (24.5)	
*Missing, n(%)*	89 (6.7)	8 (7.3)	

*Abbreviations*: *IQR* Interquartile range, *SD* Standard deviation, *HbA1c* Glycosylated hemoglobin, *eGFR* Estimated glomerular filtration rate, *ACR* Albumin/creatinine ratio in urine, *BMI* Body mass index, *GLP1* Glucagon-like peptide-1, *SU* Sulfonylurea, *SGLT2* Sodium-glucose transport protein 2, *DPP4* Ddipeptidyl peptidase-4

Specialist letters were available for 94 out of 110 patients treated in secondary care (85.5%) (Table 3). The reason for referral was known in 81 patients (73.6%) and in 64 patients (79.0%) of them, it was according to the RTA. Reasons to refer patients were most often insufficient regulated HbA1c and diagnostic uncertainty (Table 4). Of all patients treated in secondary care 80 patients (72.7%) had a profile that matched eligibility for treatment in secondary care according to the RTA.

#### COPD

COPD patients treated in primary care compared to those in secondary care had better spirometry results (FEV1%pred: 67.39 versus 57.53, *p* < 0.001), had a lower CCQ score (1.15 versus 2.05, *p* = 0.015) and a lower MRC dyspnea scale score (1.49 versus 2.50, *p* = 0.001). Fewer patients used inhalation medication (75.2 versus 90.1%, *p* < 0.001) and there was less polypharmacy in primary COPD patients (49.0 versus 64.1%, *p* = 0.002) (Table 2). Pulmonologist letters were available for 102 patients (84.3%) treated in secondary care (Table 3). The reason for referral was known for 72 (59.5%) of them. Reasons to refer patients were most often; diagnostic uncertainty and insufficient treatment result with the current medication (Table 4). It was not possible to determine in all patients whether the reason for the referral was according to the RTA or not. The RTA states that patients not meeting criteria for treatment in primary care should be referred to secondary care. But not all these criteria could be assessed (e.g. on exacerbation frequency due to insufficient registration). The profile of 38 patients (31.4%) matched eligibility according to the RTA criteria for treatment in secondary care.

**Table 2:** Profile of the COPD patients in primary and secondary care

**COPD**			
	**Primary care (*n* = 270)**	**Secondary care (*n* = 121)**	***p*-value**
**Sex**			
*Male, n (%)*	130 (48.3)	55 (45.5)	0.62
**Age (mean, range) in years**	67 (38–99)	67 (40–94)	0.89
**Systolic blood pressure (mmHg, mean ± SD)**	133 ± 14.87	132 ± 16.50	0.62
*Missing, n*	61	37	
**Inhalation medication**			< 0.001
*Yes, n (%)*	195 (75.2)	109 (90.1)	
*No, n (%)*	71 (26.3)	12 (9.9)	
*Missing(%)*	4 (1.5)	0 (0)	
**Spirometry (percentage on FEV**_**1**_** of predicted, mean ± SD)**	67.39 ± 16.75	57.53 ± 19.14	< 0.001
*Missing, n*	126	37	
**Smoking**			0.14
*Yes, n (%)*	110 (40.7)	31 (25.6)	
*No, n (%)*	14 (5.2)	6 (5.0)	
*In the past, n (%)*	101 (37.4)	48 (39.7)	
*Missing, n(%)*	45 (16.7)	36 (29.8)	
**BMI (kg/m**^**2**^**, mean ± SD)**	26.70 ± 5.09	27.49 ± 5.52	0.30
*Missing, n*	58	62	
**CCQ (mean ± SD)**	1.15 ± 0.73	2.05 ± 0.66	0.015
*Missing, n*	101	117	
**MRC (mean ± SD)**	1.49 ± 1.03	2.50 ± 1.29	0.001
*Missing, n*	142	107	
**Frailty**			0.11
*60 years or older (n)*	196	92	
*Yes, n (%)*	115 (58.7)	68 (73.9)	
*No, n (%)*	34 (17.3)	11 (12.0)	
*Missing, n*	47 (24.0)	13 (14.1)	
**Polypharmacy**			0.002
*60 years or older (n)*	196	92	
*Yes, n (%)*	96 (49.0)	59 (64.1)	
*No, n (%)*	52 (26.5)	10 (10.9)	
*Missing, n(%)*	48 (24.5)	23 (25.0)	
**Multimorbidity**			
*Yes, n (%)*	214 (74.3)	86 (71.1)	0.22
*No, n (%)*	46 (16.0)	26 (21.5)	
*Missing, n(%)*	28 (9.7)	9 (7.4)	

*Abbreviations*: *SD* Standard deviation, *FEV*_*1*_ Forced expiratory volume in 1 s, *BMI* Body mass index, *CCQ* Clinical COPD Questionnaire, *MRC* Medical Research Council dyspnea scale

**Table 3:** Information about patients referred to secondary care

	**Diabetes Mellitus type 2** **Secondary care**	**COPD** **Secondary care**
**Letter with information from secondary care in the study period**		
*Total patients, n (%)*	110 (100)	121 (100)
*Yes, n (%)*	94 (85.5)	102 (84.3)
**Reason for referral to secondary care known**		
*Total patients, n (%)*	110 (100)	121 (100)
*Yes, n (%)*	81 (73.6)	72 (59.5)
**Reason for referral according to RTA when the reason for the referral is known**		
*Total patients, n (%)*	81 (100)	72 (100)
*Yes, n (%)*	64 (79.0)	**Not possible for COPD**
**Does the profile of the patient fit in secondary care using data from 2017 until 2019**		
*Total patients, n (%)*	110 (100)	121 (100)
*Yes, n (%)*	80 (72.7)	38 (31.4)

**Table 4:** Reasons to refer a patient to secondary care, when the referral reason is known

**Diabetes Mellitus type 2**	**COPD**
Insufficient glycosylated hemoglobin regulation	Dyspnea (on exertion)
T2DM in combination with pregnancy wish	Coughing
Patient wishes a sensor indicator/ insulin pump/ glucose flash monitoring	Exacerbation of COPD
Diagnostic uncertainty on type 2 diabetes	COPD ‘The Global Initiative for Chronic Obstructive Lung Disease’ stage 3 or 4 (fitting with FEV1% < 50%)
Starting with a glucagon-like peptide-1 agonist	Pneumonia combined with COPD
Side effects of the medication	Insufficient treatment with the current medication
Elevated albumin/creatinine ratio in the urine (for advice)	Inconclusive spirometry
Neuropathic pain	Both obstructive and restrictive lung disease
Patient wishes a referral	Patient wishes a referral
The need for a new referral to secondary care (patient went to secondary care in the past)	Diagnostic uncertainty ons COPD
Hypoglycemia	Both COPD and asthma
Macrovascular complications	High burden of disease

## Discussion

The results of the current study showed that most of the primary care patients were treated at the right place according to the RTA. Besides, those who were correctly treated in primary care had less complex T2DM or COPD compared to those correctly treated in secondary care.

An important strength of our study is that we had a dataset with data from patients in both primary and secondary care, which made it possible to analyze these the profiles of both patient groups. It was difficult to determine if patients were correctly treated in secondary care mainly for the COPD patients, due to the time range of available data (2017–2019). For example, referral letters to secondary care of more than two years ago were missed, resulting in incorrect labeling of correct treatment setting of a patient. Therefore, conclusions about the number of correctly treated patients in secondary care should be made with caution. Another limitation is that in the current study, data on exacerbations for COPD patients was missing, due to insufficient registration. Therefore the RTA criterium to refer a patient to secondary care if a patient experienced two or more exacerbations in the last year could not be assessed.

Nationwide the prevalence for T2DM is 5.3% and for COPD 3.4%, in our study for DM 3.3% and for COPD 0.9% [1, 2]. In Europe, the prevalence of T2DM is 10.2% in men and 8.5% in women [18]. The worldwide prevalence of COPD varies from 8 to 10% [19]. The overall prevalences found in this study are lower than nationwide, this might be due to the location of the primary care practices; a newly developed residential area with a younger population. Nationwide percentages of patients treated in secondary care are 9.5% for T2DM and 27.2% for COPD [3, 4]. Our results for the percentage of patients treated in secondary care seem comparable to the nationwide data.

The profile of patients treated in primary and secondary care, are in line with another Dutch study including T2DM patients. This previous study also found that patients in secondary care had a longer diabetes duration, used more insulin, had a higher prevalence of complications, a higher BMI and higher HbA1c levels [20].

In this study patients with T2DM with an indication for using a glucagon-like peptide-1 receptor agonist were still referred to secondary care. Currently T2DM patients are no longer referred for this indication, and glucagon-like peptide-1 receptor agonist can now be prescribed by the GP. Therefore, it is expected that the number of patients treated in secondary care based on this indication will decrease. The question remains if patients currently treated in secondary care with this indication will also be referred back to primary care and if al GPs feel qualified enough to prescribe and treat patient with glucagon-like peptide-1 receptor agonist.

With regard to the COPD findings, these are comparable to a previous Dutch study which also found significant worse spirometry measures for their secondary (and tertiary) care population compared to the primary care populations [21]. Furthermore, the primary care population also had the least severe degree of airflow limitations (fitting with the RTA criterium on FEV1%), the least symptoms in daily life and the best functional status. In contrast to our findings their secondary care population was significantly older and had the highest comorbidity score [21]. Our study showed less symptoms and better functional status in primary care since scores on CCQ and MRC dyspnea scale were significantly lower. This is in line with what is desired to achieve with the RTA.

## Conclusions

In conclusion, the current study found percentages of patients with T2DM and COPD treated in secondary care that are in line with national percentages. There are differences in the profile of patients in primary versus secondary care in both T2DM and COPD on various characteristics, including age and HbA1c for T2DM and the use of inhalation medication and spirometry results for COPD. Patients with a better condition were treated in the primary care setting. Of the T2DM patients treated in secondary care, the majority were correctly treated there, this percentage was lower for COPD patients.

We recommend further research to determine if patients with T2DM and COPD are referred back to primary care when healthcare in secondary care is no longer indicated based on the RTA and the project ‘The right care at the right place’. If patients are not referred back, reasons for this need to be explored, so the RTA and care for patients can be improved.

## Supplementary Information

**Additional file 1.** Reasons to treat patients in secondary care according to the RTA.

## Data Availability

The datasets used during the current study are available from the corresponding author on reasonable request.

## Abbreviations

DM: Diabetes Mellitus
COPD: Chronic Obstructive Pulmonary disease
T2DM: Type 2 Diabetes Mellitus
RTA: Regional Transmural Agreements
ICPC: International Classification of Primary Care
IQR: Interquartel range
HbA1c: Glycosylated hemoglobin
CCQ: Clinical COPD Questionnaire
MRC: Medical Research Council
GP: General Practitioner
