# Supplementary material for: Type 2 diabetes and COPD: treatment in the right healthcare setting? An observational study
Source: BMC Fam Pract. 2021 Apr 20;22:78. doi: 10.1186/s12875-021-01424-w (PMC8059302; doi:10.1186/s12875-021-01424-w)
Supplement: Supplementary file 1 — Additional file 1. Reasons to treat patients in secondary care according to the RTA. [file 12875_2021_1424_MOESM1_ESM.docx]

# Appendix I - Reasons to treat patients in secondary care according to the RTA

**RTA T2DM**

**Indications for referral of patients to secondary care:**

- Diagnostic uncertainty on thetype 2 diabetes
- Insufficient T2DM regulation
- Insulin use

- By (professional)drivers

- Combined with worries about increasing weight or about using insulin

- Recurrent hypoglycemia

- Inadequate T2DM regulation when using insulin on a one or two times a day scheme

- Imminent dehydration or hyperglycemia
- Serious hypoglycemia when using long-acting medication
- Persisting LDL-cholesterol higher than 2,5 mmol/l combined with a high-risk profile
- Total cholesterol higher than 8 mmol/l, cholesterol ratio higher than 8 or LDL-cholesterol higher than 5 mmol/l (referral after checking for pregnancy, alcoholism and hypothyroidism)
- Fasting triglycerides higher than 6 mmol/l
- Hypertension (with a systolic blood pressure higher than 140 mmHg in patients younger than 80 years and a systolic blood pressure higher than 160 mmHg in patients older than 80 years despite triple therapy)
- Obesity that is causing health risks
- Patients younger than 65 years with an estimated glomerular filtration rate of 45 to 60 ml/min or patients older than 65 years with an estimated glomerular filtration rate of 30 to 45 ml/min (referral for advice)
- Patients younger than 65 years with an estimated glomerular filtration rate below 45 ml/min or patients older than 65 years with an estimated glomerular filtration rate below 30 ml/min (permanent referral)
- Increase of microalbuminuria
- Diabetic foot ulcer
- Neuropathy
- Pregnancy or pregnancy wish

**RTA COPD**

**Circumstances when to treat patients preferably in primary care:**

- A forced expiratory volume in one second of more than 50% of predicted or more than 1.5 liters
- A stable forced expiratory volume (FEV1%)
- A score lower than three on the Medical Research Council (MRC) dyspnea scale
- A score lower than two on the Clinical COPD Questionnaire (CCQ)
- A body mass index higher than 21
- A stable body mass index (less than 5% weight loss in a month or less than 10% weight loss in six months)
- Fewer than two exacerbations a year in whichoral corticosteroids were necessary
- 'Limited comorbidities'
